# Neuro-symbolic reasoning engine for tax optimisation

**DOI:** 10.3389/frai.2026.1802755

**Published:** 2026-05-07

**Authors:** Karthika Veeramani, Allen Joseph N, Pavithran M

**Affiliations:** 1 School of Computer Science and Engineering, Vellore Institute of Technology, Chennai, India; 2 School of Electronics Engineering, Vellore Institute of Technology, Chennai, India

**Keywords:** explainable AI, hybrid AI systems, Indian Income Tax Act, knowledge graphs, large language models, legal reasoning, neuro-symbolic AI, tax optimization

## Abstract

Automating tax calculations and optimisation is challenging because modern AI systems such as large language models operate probabilistically, while legal and financial reasoning requires deterministic compliance with statutory rules. This research presents a new approach to automate taxes in India through the Neuro-Symbolic Tax Optimizing Engine (NTOL) that combines the advantages of large language models (LLMs) and a deterministic symbolic reasoning component based on a structured knowledge graph of tax statutes. This research builds on prior work in neural semantic parsing, combining neural semantic parsing with formalized symbolic rules that are immutable ensuring that output complies with the law, producing reproducible calculations and being independently auditable. The system was evaluated using a curated benchmark dataset consisting of 20 tax computation scenarios derived from provisions of the Indian Income Tax Act, 1961, including business income adjustments, deductions under Sections 80C and 80D, and presumptive taxation under Section 44AD. The proposed approach was compared against two benchmarks, an LLM only system and with a simple Retrieval-Augmented Generation (RAG) system. Evaluation results indicate that the proposed approach outperformed both the benchmarks across multiple performance characteristics including improved accuracy, compliance with legal statutes, robustness to complexities within legal statutes and support for explainable results. In particular, in contrast to the LLM-only system, which achieved a 75% accuracy, and the simple RAG system, which only achieved a 60% accuracy; the proposed system achieved an accuracy of 80%. Most of the inaccuracies were due to incomplete knowledge graph coverage rather than hallucinated reasoning, whereby the symbolic layer limits non-valid computation paths and does not produce unsupported output when the system lacks an output. In addition, the system's performance was analyzed using qualitative methods such as determining semantic completeness and reasoning consistency. These qualitative methods provide an assessment that demonstrates the system generates computations that are consistently maintainable and auditable, therefore reducing inconsistencies, such as artificial intelligence hallucinations; this provides tremendous value to stakeholders through the provision of transparent computation trails that facilitate audit readiness, reduce the amount of time required for compliance verification, and help to mitigate the risk of litigation due to incorrect tax calculations.

## 1 Introduction

According to Bhuyan et al. (2024), Artificial Intelligence has two major research areas, namely NLP and Symbolic Reasoning, which together represent a new way for AI systems to understand and structure knowledge as well as to reason about more complex domains such as Law, Finance, and Governance. The reasoning accuracy in the Legal and Finance decision-making domains must be strictly adhered to because the output from the AI system has to meet a formally defined regulatory framework. One of the most logistically challenging domains to automate with artificial intelligence is tax computation and optimisation (Saragih et al., 2023). This is due to the reason that tax computation and optimisation requires a high level of logical interdependence between a series of conditions or exceptions and also due to the fact that tax law is subject to change on a frequent basis through amendments. The Indian Tax ecosystem, governed by the Income Tax Act, 1961, and all subsequent updates, constitutes a large and complex knowledge domain, containing many provisions, exceptions, conditions, and cross references that are highly interdependent upon one another. Historically, digital tools that have been created for the purpose of performing tax computation generally rely upon static rule-based approaches to encode the logic contained in the various Indian tax laws.

While these systems can be configurable to comply with Indian tax laws to some extent, they are also extremely static, rigid, and do not scale well in a logical sense, nor do they provide support for the contextual or semantic interpretation of natural language inquiries. Conversely, the introduction of large-scale language-models in the form of GPT-4 and soon, GPT-5, represent tremendous advancement in the area of NLP, including improvements in the areas of textual comprehension, semantic extraction, and contextual reasoning. Nonetheless, at the same time these systems experience built-in weaknesses, including hallucination, little ability to clearly articulate reasoning, and being probabilistic, therefore possibly presenting contradictory answers that conflict with existing prescriptive rules (Saba, 2023). As a result, LLMs cannot be used practically in industries where adherence to a prescribed set of rules is imperative to establish logical and verifiable conclusions, such are found in finance and law (Zubaer et al., 2023). In practice, errors in tax computation can impose large economic expense on firms through penalties, compliance audits, and the a lot of professional time required for manual verification of tax obligations. Thus, by merging these two technology paradigms, symbolically defined logic and advanced deep learning neural networks, it is possible to create a more powerful framework by which to reason accurately while being transparent, adaptive, and contextually aware (Zhu et al., 2023).

Even with the advances in legal AI and computational reasoning, machine learning systems and other computational reasoning systems typically struggle with successfully achieving a balance between interpretability and adaptiveness (Górski et al., 2025). For example, traditional symbolic systems allow users the ability to be confident that their results were generated through a logically correct chain of reasoning but do not adequately account for the ambiguity of language typically found in natural language documents, such as laws and contracts. In contrast, traditional neural networks can generalize across different forms of language and create machine-generated results, but their responses do not necessarily reflect true legal truth in all cases (Wei et al., 2025). In order to develop a way to bridge the gap between these two extremes, researchers have increasingly turned to hybrid approaches that take advantage of the unique strengths of each type of system (Cunnington et al., 2023). By combining the ability of neural semantic representation with a structured approach to logical reasoning, hybrid systems provide for an ability to have explainable decisions based both on empirical data and on a logical basis.

Researchers have found that such hybrid approaches in other fields such as biomedical informatics (García-Barragán et al., 2025), automated compliance and policy interpretation, and compliance automation have the potential to significantly decrease the number of factual errors, increase the level of interpretability, and enable users to make dynamic updates to their knowledge base. However, applying the same hybrid methodology for calculating taxes presents unique difficulties, particularly when it comes to India, due to the highly interconnected and hierarchical nature of the Indian tax code and the fact that tax codes are updateable and subject to change on an ongoing basis (Zhong et al., 2024). The establishment of these relationships necessitates the understanding of the relevant language, the capability to construct, conceptualize, maintain, and manipulate the relationship between the legal entities, dependencies, and symbolic rules. As such, the intersection of symbol-based reasoning with LLMs is a complex yet promising territory for the automation of tax computation and optimizing taxes in a manner that is legally valid and interpretive (Yu et al., 2020).

Even with those improvements, a significant portion of the current hybrid system gap exists where the current procedures do not provide an adequate way to interpret the numerous provisions of a statutory legal framework and the numerous conditional clauses and cross-references that must be found within such frameworks at the same time (e.g., with the Indian Income Tax Act). The purpose of this research is to examine how a combination of neural and symbolic reasoning can be used to improve the accuracy of calculating taxes based on the Indian Tax Act through logical consistency and clarity (Yang et al., 2025). It will investigate ways to combine the information contained within a representation of the Indian tax law as a “knowledge graph” with the ability of a LLM to reason within the rules of that tax law (Yang et al., 2024). In particular, it will develop a system capable of understanding tax-related questions presented in natural language, reasoning through structured legal documents stored in a database of tax law, and providing answers that provide contextually accurate responses that meet all of the requirements dictated by the law. Consequently, this research will attempt to answer three specific questions:

1) How can a hybrid neuro-symbolic framework adequately reflect and process the data and relationships found within the Indian tax laws?
2) What techniques will be used to ensure that the tax reasoning provided by LLMs will be grounded in fact, free from false information, and consistent with the law?
3) What impact will the use of both symbolic rules and neural reasoning have on the level of accuracy, interpretability, and flexibility of tax optimisation systems?

The rest of the paper is organized as follows: Section 2 provides an overview of past research conducted on Neuro-Symbolic Reasoning and Tax Automation, outlining any challenges in previous studies due to limitations with regards to both motivation and methodology. Section 3 provides detailed information on the proposed architecture, including construction methods of knowledge graphs and the symbolic rule engine's functionality. In Section 4, the evaluation strategies employed to determine system performance and system accuracy are presented. Section 5 describes both the limitations of our study and proposed directions for future research. Finally, Section 6 summarizes the major contributions and conclusions of this research.

## 2 Related works

Tax prediction and automation technology has made great advances by combining new technologies such as knowledge graphs, deep learning and symbolic reasoning to allow for intelligent decision making in the area of taxes. The findings of recent studies indicate that both interpretability and computational accuracy are necessary for effective taxation reasoning systems and that these two characteristics cannot be produced using either pure neural networks or rule based systems in isolation. Knowledge Graphs (KGs) provide an extensive and structured representation of legal knowledge, while large language models (LLMs) provide semantic understanding and adaptability. Nonetheless, the findings of these studies indicate that the legal and tax reasoning functions require deterministic output, traceability and strict compliance with regulatory standards which cannot be provided by neural systems alone. Therefore, New Hybrid Neuro-Symbolic Systems that merge the learning and analysis capabilities of data driven systems with the verification capabilities and explainability of rule-based systems have proven to be a powerful solution. The research in these studies provided the basis for the proposed Neuro-Symbolic Reasoning Engine for Tax Optimisation, which uses GPT-5 Mini for the purposes of extraction and explanation, Neo4j for structured storage, and a rule-based symbolic layer to ensure a strict ground truth and authoritative inference. The following survey positions the proposed architecture within this evolving research landscape by presenting an integrated view of the literature across seven dimensions: tool-centric, algorithmic, deep-learning, synthesis, declarative KG, evaluation centric and hybrid frameworks.

There is substantial research demonstrating the need to have strong and traceable engineering pipelines in any automated tax or legal solution. For example, Intuit's Tax Knowledge Graph and Fintech Analytics Systems illustrate how ingestion pipelines, provenance tracking and version control should be considered primary design decisions, not just implementation details. Systems that rely on this approach use various methods including conservative text segmentation using regex/OCR, automated extraction of entities and relationships through the use of LLMs, and canonicalization of node identifiers to ensure semantic consistency. For this reason, many applications that use Neo4j also use vector indexes to provide both deterministic and similarity-based querying. The architecture pipeline proposed segmentation of the Income Tax Act 1961 using regex, triple extraction via GPT-4o mini, storage in Neo4j and attaching provenance metadata illustrates an example of applying these fundamental design principles within the context of this project. By also adding symbolic rules as immutable constraints, the proposed solution enhances the basic architecture of previous work. Significant operational trade-offs discussed in the literature, including latency due to RAG retrieval, telemetry overhead, rule governance and compliance, directly tie to the challenges addressed by the proposed system through caching, provenance logging and human-in-the-loop validation (Yu et al., 2020; Pahsa, 2024).

There has been significant, focused work performed in algorithmic research for improving Knowledge Graph (KG) strength and depth of reasoning, primarily through representation learning and the incorporation of rules. Models such as TransE, RotatE, and BoxE use distributional embeddings, along with logical regularization, to retain the relational semantics of each unit in the relation space (Spillo et al., 2024). They also provide multi-hop reasoning due to the ability to calculate a loss function effectively using the rules extracted from knowledge sources. These rules, which are based on the tax conditions (e.g. whether the person lives there, how much money they make, and what types of investments they have), yield a confidence score as they relate to tax eligibility.

Additionally, work being done in Inductive Logic Programming (ILP) and Feed-Forward Neuro-Symbolic Learning (Cunnington et al., 2023) provides examples of how learned rules (i.e. mined rules) can act as strict auxiliary constraints to increase both retrieval precision and interpretability. In contrast to the potential value provided by mined or learned rules, much of the literature on legal reasoning cautions about the potential problems associated with mined or learned rules overriding curated/human validated rules (Spillo et al., 2024; Cunnington et al., 2023). Previous research provides further support for the operationalisation of this characteristic in our proposed system as we would consider the symbolic rules as “absolute ground truth,” and we would leverage the learned relational rules largely for context enrichment purposes. This also aligns with the best practice for algorithmic hybrid KG reasoning. The addition of uncertainty scoring and path-confidence metrics to the retrieval logic further aligns with the best current/research strategies for retrieval logic in the domain of probabilistic KG reasoning (Spillo et al., 2024).

Deep learning and LLMs-based approaches have performed very well in Natural language generation and Information extraction; however, their capability to do reporting-like legal reasoning is limited by being deterministic. Attention has been given to LLMs targeting tax-oriented queries: the research (Zhong et al., 2024) on ChatGLM-6B's performance after fine-tuning on answering tax questions and VITA benchmarking of both GPT-4 and Llama (Gogani-Khiabani et al., 2025). However, both studies have shown that LLMs tend to make mistakes when performing mathematical calculations and their understanding of the clauses being used in questions. Therefore, some researchers agree that there is a division of responsibilities between LLMs and symbolic engines, with LLMs used for semantic extraction, paraphrasing, and human facing life explanations while symbolic engines enforce numerical accuracy and legal accuracy (Zhong et al., 2024; Gogani-Khiabani et al., 2025; Bhuyan et al., 2024). This methodology aligns with the proposed architecture since the symbolic module will provide the authoritative reasoning, while the GPT-4 Mini will be used to do the Extraction and Synthesis pieces. The literature also argues against embedding the legal constraints into the model's weights directly due to the continual evolution of statutes, which requires frequent, explicit, and inspectable updates (Wei et al., 2025; Górski et al., 2025). By having the normative logic externalized in symbolic rules rather than fine-tuning the model, the present design adheres to the best approaches for building Legal AI systems that are transparent and also maintainable.

The employment of declarative computation in conjunction with tax knowledge graphs is significant because it enhances user-explanation of its accuracy in terms of reasoning. For example, Intuit's TurboTax Knowledge Graph (Yu et al., 2020) consists of two types of subgraphs: a “Calculation Graph” that includes all of the numeric dependencies, and a “Completeness Graph” used to validate whether someone has met all of the requirements to file their taxes. This implementation provides both computational determinism and the ability to demonstrate how calculations are derived back through the tax code for explanations of results. Documented work in the areas of explainable AI specific to taxation and law (Yu et al., 2020; Górski et al., 2025) indicates that all recommendations and advice should allow the user, as well as the Auditor, to derive back to its statutory base.

Neo4j will be used for the storage of factual data with added provenance metadata and with the possibility of including executable “calc nodes” to enable deterministic mathematical reasoning associated with these data. These components provide compliance to the industry expectations for managing this information. Procedural nodes will be embedded within the graph to provide formulae for deduction limits, etc., giving the proposed system the ability to conduct symbolic computation natively, rather than having to rely on the arithmetic capabilities of LLMs. Achieving this capability will result in greater transparency and replication of the results. As a result, the current design is expected to enhance the rationale power of the declarative KGs and enhance the ability to construct a neuro-symbolic synthesis from the two approaches.

The previous research examining a wide array of sub-fields is appropriate for developing the proposed Neuro-Symbolic Tax Optimization Engine. Studies on tools and platforms (Yu et al., 2020; Pahsa, 2024) focus on providing the necessary infrastructure to ingest knowledge graphs (KGs) through provenance; studies on algorithm (Zhu et al., 2023; Spillo et al., 2024) focus on a procedure for retrieving information that is confidence-aware and for producing rule-regularized embeddings; studies in deep learning (Zhong et al., 2024; Gogani-Khiabani et al., 2025; Wei et al., 2025) identify the scope of boundary roles for LLMs; and neuro-symbolic studies (Tong and Hu, 2024) provide a rationale for combining symbolic and neuro components in a real-time environment. Research on declarative computation (Yu et al., 2020) supports the proposed method of embedding procedural components of tax optimization into the KG; and studies on evaluation (Lorello et al., 2025) support the decision to include both provenance and rule governance as additional components of the proposed system. As such, the proposed system combines knowledge obtained from these various areas of multidisciplinary and applied research into a single cohesive framework, with the following three distinct components establishing a legally auditable and computationally valid framework for tax optimization under the Indian Income Tax Act: (1) GPT-5 Mini for extraction and justification, (2) Neo4j as the structured form of knowledge, or KD, for the KG, and (3) The symbolic reasoning layer for deterministic inference.

The legal and tax fields are exploring variations of autonomous evaluation through hybrid approaches using AI systems instead of relying on only standard metrics (e.g., accuracy, f1) to evaluate success or failure of AI in these fields, which place a high priority on explainability, traceability, and legal defensibility (Zhong et al., 2024; Gogani-Khiabani et al., 2025). The literature has identified similar issues associated with AI in these fields that may affect compliance or lead to potential non-compliance (for example: mathematical errors; retrievals that are not legally relevant; and digital drift as a result of unsupervised rule-building) (Gogani-Khiabani et al., 2025; Górski et al., 2025). To mitigate these types of compliance issues, AI systems are designed with a combination of extraction confidence; provenance tracing; and manual adjudication checkpoints. The design of the system incorporates all three areas by implementing symbolic rule precedence; provenance tracking; and a manual validation loop, all of which will help ensure credibility of evaluation standards for the proposed architecture, ensuring the architecture contains equal parts of reliability, explainability, and numerical accuracy.

Hybrid approaches to the combination of neural and symbolic reasoning allow for the development of two-way feedback loops between a neural network and a symbolic representation, producing results that are both interpretable and legally compliant. Foundational work on hybrid approaches to legal reasoning (Bhuyan et al., 2024) describes two-way pipelines between an LLM and a symbolic representation (as well as NeSy loops) to minimize hallucination and facilitate easy auditing. Canonicalisation, synonym normalization, confidence gating, and strict precedence of rules are the techniques used in these hybrid models to provide correct and deterministic answers. Thus, the Neuro-Symbolic Tax Optimisation Engine merges these principles by employing curated rules of symbolic legal authority as the primary knowledge source with respect to any knowledge inferred by an LLM, thereby establishing a clear hierarchy of authoritative sources. Additionally, provenance connections and entity normalization (for example, resolving “assessee” versus “individual”) preserves terminological consistency, and Neo4j traversal and symbolic verification work in concert to enable both semantic and logical retrieval. Finally, literature identifies continuous co-evaluation, which occurs when verified corrections to the symbolic representation of legal knowledge are incorporated back into the knowledge graph to improve future extraction from the LLM; this co-evaluation process can extend naturally from the proposed architecture (Bhuyan et al., 2024).

### 2.1 Motivation

The Indian taxation system's elaborate nature and rapidly changing tax structure often pose a significant obstacle for many individuals and businesses seeking optimal means to prepare for taxation, thereby resulting in improper preparations. The Income Tax Act of 1961 after its numerous amendments contains an extremely convoluted, interconnected, conditional and exempt structure. Legal experts who rely primarily upon the interpretation of tax rules encounter great difficulty in understanding the intricate nuances of their meanings within the legal context. Furthermore, an entirely neural network approach i.e., a “LLM” does not provide for the logical consistency, explainability or compliance that are necessary in tax.

**Figure 1:**
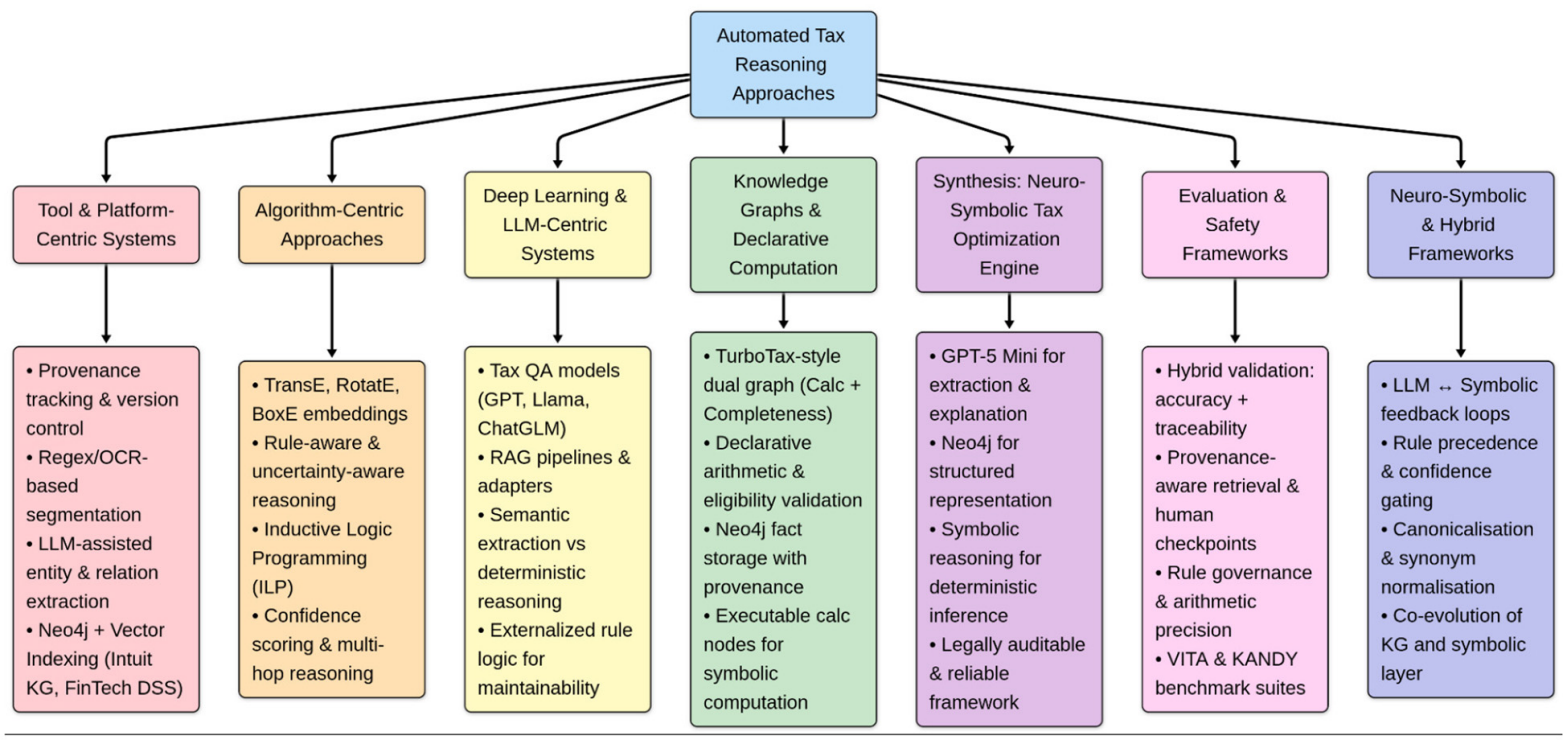
Summary of literature survey.

The impetus for this research, as shown in Figure 1 was to build an intelligent, dependable, and machine-readable tax reasoning model that utilizes established statutory principles, goals and procedures for tax purposes by integrating the legal correctness of tax reasoning through symbolic logic with the flexibility of natural language interpretation built upon neural networks into a single neuro-symbolic reasoning engine. A neuro-symbolic reasoning engine is capable of overcoming the shortcomings associated with both endpoints based solely upon the rules of law i.e., Expert Systems and endpoints based upon probability i.e., LLMs. The main aim of this research is to build a system that provides a transparent, automated means of optimizing tax, while minimizing manual input and maximizing the ability to produce accurate and interpretable outcomes in financial decision-making.

### 2.2 Research challenges

Creating a neuro-symbolic reasoning engine for optimizing tax liability using the Indian taxation system entails numerous interrelated issues related to research. The primary discussion point refers to managing the complexity and interdependence of the language of statutes, including the Income Tax Act, which employs numerous cross references, exceptions and conditional clauses. The research involved constructing a knowledge graph based on unstructured legal text by successfully extracting the relevant entities and relationships from the text, eliminating any redundancy and maintaining semantic consistency of the entities within the context of the Income Tax Act. The research process for constructing the knowledge graph is characterized by a prolonged process of approximately eight hours of iterative extraction and validation of the extracted entities using a large language model. The time required to complete the extraction process, including repeating and validating the required contextual information, illustrates the difficulties inherent in converting the language of statutes into a structured, machine-interpretable format. The integration of neural and symbolic components was a significant challenge during the research. The user queries were significantly more difficult to parse than the previously listed features since the user would have been expected to submit requests in a natural language format. As a result, once the natural language input has been parsed, it needs to be successfully mapped to the appropriate and relevant knowledge node(s) in the knowledge graph. Also complicating matters is that the system must provide logical consistency and also be able to explain itself while avoiding contradictions between the neural approaches and traditional symbolic rule-based methods. The final consideration is whether the proposed system will work effectively at scale, e.g., if context updates are common due to the rapid pace of change in legislation, then it is imperative to accommodate those increased demands while still ensuring the same level of accuracy and coherence. Thus, these challenges represent the technical and conceptual basis upon which this investigation is grounded.

### 2.3 Contribution

The contribution made by this work to the area of neuro-symbolic reasoning and automation of taxation is based on a hybrid model which can be applied in compliance with Indian Tax Law.

1. A Knowledge Graph creation framework is designed for the Income Tax Act 1961 using a LLM to extract the entities, attributes and relationships, providing structured and interpretable representations of tax law, and the interdependencies among various laws.
2. A Hybrid Neuro-Symbolic Reasoning pipeline that incorporates both expert defined tax law with symbolic constraints and LLM reasoning to ensure compliance while also taking advantage of the generative features of a LLM to provide context and provide natural language responses post computation of tax.
3. A Two Layer Querying Framework where hub queries retrieve semantically similar elements from the Knowledge Graph and then combine those with relevant statutory factors to provide accurate, relevant and meaningful tax advice for tax advisory systems using LLM technology in accordance with the Income Tax Act of India.

### 2.4 Novelty

Research in this area, as shown in the Table 1 combines the principles of neuro-symbolic reasoning with the use of data from knowledge graphs to provide a method for automating tax optimisation for the benefit of taxpayers under the relevant legislation. Unlike the typical LLM approach of answering queries based upon only probability, the proposed approach integrates various forms of learnt knowledges as well as the use of symbolic rules/facts where they exist, i.e., to establish both a logic backbone for correct use and a method of integrating the facts contained within the relevant Law.

**Table 1:** Comparative analysis of automated, deep learning, and neuro-symbolic approaches for tax and legal reasoning based on the provided bibliography.

Category	Title/reference	Year	Methodology	Dataset and domain	Performance metrics
Tool & Platform-centric systems	Tax Knowledge Graph for a smarter and more personalized TurboTax (Yu et al., 2020)	2020	Dual-graph (calculation + completeness) architecture for explainable tax computation	TurboTax platform, U.S. tax datasets	Deterministic reasoning, auditability
	Financial technology decision support systems (Pahsa, 2024)	2024	Decision support frameworks using big data and KG for financial compliance	Financial institutions, taxation modules	System efficiency, decision accuracy
Algorithm-centric approaches	Predicting tax defaults through feature transformation and XGBoost (Waiker et al., 2025)	2025	Feature transformation with XGBoost optimization for default prediction	Taxpayer default records	F1 score, AUC-ROC, Accuracy
	TGR: Neural-symbolic ontological reasoner for domain-specific KGs (Zhu et al., 2023)	2023	Ontology-based reasoning over domain-specific knowledge graphs	Domain-specific KGs	Reasoning precision, hit ratio
Deep learning and LLM-centric systems	Tax Intelligent Decision-Making Language Model (Zhong et al., 2024)	2024	LLM-based tax QA and decision support	Public tax question datasets	Accuracy, explainability score
	Performance of LLMs on VITA test: AI-assisted tax returns (Gogani-Khiabani et al., 2025)	2025	Evaluation of LLMs on low-income taxpayer assistance scenarios	VITA benchmark dataset	Arithmetic accuracy, clause recall
	Automated attribute extraction from legal documents using LLMs (Adhikary et al., 2026)	2024	Zero-shot and few-shot extraction from legal corpora	Legal documents, statutory texts	Precision, Recall, F1 score
Hybrid neuro-symbolic frameworks	An LLMs-based neuro-symbolic legal judgment prediction framework (Wei et al., 2025)	2025	Integration of LLMs with symbolic logic for civil case prediction	Civil case legal documents	Judgment accuracy, logical consistency
	Neuro-symbolic artificial intelligence: a survey (Bhuyan et al., 2024)	2024	Comprehensive review of neural and symbolic integration strategies	Multi-domain applications	Model interpretability, scalability
	Future of education with neuro-symbolic AI agents (Tong and Hu, 2024)	2024	Self-improving adaptive instructional systems using hybrid AI	Educational datasets	Adaptation speed, learner accuracy
Knowledge graph and declarative systems	A review on synergizing knowledge graphs and large language models (Yang et al., 2025)	2025	Review of KG-LLM synergy for improved factual grounding	General and domain KGs	Factual consistency, hallucination rate
	Recommender systems based on neuro-symbolic KG embeddings (Spillo et al., 2024)	2024	Encoding first-order logic rules into KG embeddings	E-commerce/ recommendation datasets	Mean Reciprocal Rank (MRR), Hit Rate
Evaluation and benchmarking	The KANDY benchmark: Incremental neuro-symbolic learning (Lorello et al., 2025)	2025	Benchmarking reasoning capabilities with Kandinsky patterns	Visual-symbolic reasoning tasks	Incremental learning efficiency
	Exploring explainable AI in the tax domain (Górski et al., 2025)	2024	Framework for assessing XAI applicability in fiscal law	Tax administration cases	Trust metrics, explainability
	Stochastic LLMs do not Understand Language (Saba, 2023)	2023	Critique of LLMs favoring symbolic and ontological grounding	Linguistic reasoning benchmarks	Logical soundness, explainability
Synthesis/system alignment	Proposed neuro-symbolic tax optimization engine (this work)	2026	LLM extraction with Neo4j KG and symbolic enforcement	Indian Income Tax Act, 1961	Compliance rate, interpretability

This hybrid method also combines three significant innovations:

1. A semi-automated pipeline for building a knowledge graph to extract the legal entities, relationships and attributes contained in the Income Tax Act 1961, with automated refinement to remove redundancy and overlapping semantics, that is, GPT-5-mini.
2. A symbolic rule enforcement layer that is the logical underpinning of the automated tax optimisation method. This ensures that statutory requirements (laws/legislation) are used as the basis for the results of any probabilistic/Learning Language Modeling (LLM) based methods, even if the LLM based method also produces results based on probability.
3. A contextual retrieval and reasoning mechanism, whereby the knowledge graph can be queried based on the words and phrases extracted from user requests, merging the nodes with huge amounts of information/content retrieved and the necessary symbolic rules as part of the context for a natural language generator.

Prior research has examined knowledge graphs independently, LLM-based tax reasoning independently, and neuro-symbolic methods as separate entities, but the proposed framework combines all three components into one system as per the laws in the Indian Income Tax Act. Existing systems are primarily designed to operate independently of one another such that they provide only neural question answering, symbolic reasoning, or knowledge graph representations in their entirety; however, the Neuro-Symbolic Tax Optimizing Engine integrates LLM semantic extraction, structured knowledge graph representations of statutory provisions, and rule enforcement through use of symbolic rules to facilitate legally compliant tax calculations and compliance. The hybrid approach to automated tax optimisation introduces an obvious, definitive, and interpretative framework in which tax optimisation can be done based on the hybridization of symbolic logic and neural reasoning. It surpasses the legal (regulatory) consistency and explainability of pure neural methods and offers a scalable alternative to manual tax consultative services.

## 3 Methodology

The purpose of this research is to describe a new comprehensive framework for computing taxes and optimizing taxes under the Income Tax Act,1961, using a combination of neuro-symbolic reasoning. The new framework is designed to overcome one of the most important problems confronting LLM-based tax advisers, which is the phenomenon of hallucinations. The new framework combines deterministic symbolic methods with contextual comprehension and fluency that are obtained through the use of neural language models. In order to provide a more comprehensive and accurate way of calculating and determining taxes, this framework provides answers that are not only computationally accurate, but linguistically accurate, as well, and are easily checked, deleted, searched, and certified as compliant with the provisions of the Income Tax Act and the principles of taxation under Indian tax law.

**Figure 2:**
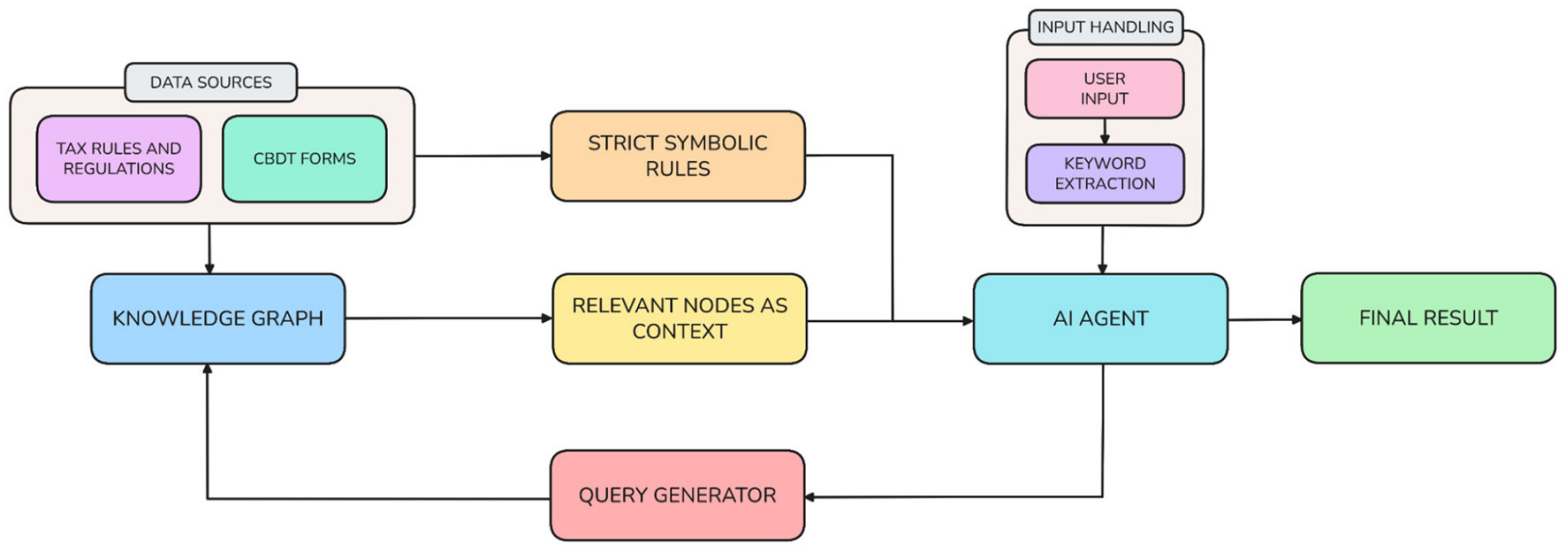
Proposed architecture figure.

The overall system design, as shown in Figure 2 for the framework consists of a collection of dependent yet independently optimized subsystems that include (1) Knowledge Graph Construction/Ingestion System, (2) Hybrid Query Processing Pipeline, and (3) Multi-Modal Response Generation System. The first subsystem creates the Income Tax Act of 1961 as an organized hierarchical and relational machine-readable representation in the form of a Knowledge Graph. The second subsystem performs complete processing of users' queries. The user's natural language is transformed into a sequential series of symbolic reasoning steps, each one producing a deterministic calculation. The third and final subsystem integrates both symbolic outputs and the associated natural language explanations into a response that can be readily understood by a human user by offering a clear picture of the basis and rationale behind the calculation of taxes.

The traditional LLM-based method for natural language processing produces text based on probabilities without validating against factual information or applying the law correctly; in contrast, the proposed method utilizes a two-fold framework, whose operational pipeline is formally described in Algorithm 1. The first layer provides factual (computationally correct) data through the defined tax code while the second layer offers the capability of being able to communicate with and understand the user's needs within a natural language interface. The combination of symbolic computing and neural computing, in addition to the separate presentation of the results of both types of computations, provides for a fully auditable outcome of the compliance with finance laws in any legal environment.

Algorithm 1 Neuro-symbolic tax optimisation pipeline.

**Require:**  User Query *Q*, Income Tax Act Documents *D*
**Ensure:**  Tax Optimization Response *R*, Audit Trail *T*
      **Phase 1: Knowledge Graph Construction (Pre-processing)**
1:  *T*_*norm*_←NormalizeText(*D*) ⊳ Remove artifacts, normalize references
2:  *E*_*raw*_←LLM_Extract(*T*_*norm*_) ⊳ Extract Entities, Attributes, Relations
3:  *G*_*KG*_←RefineAndStore(*E*_*raw*_) ⊳ Deduplicate and store in Neo4j
     **Phase 2: Hybrid Query Processing**
4:  *I, P*←IntentParser(*Q*) ⊳ Identify Intent *I* and Parameters *P*
5:  if *I* is Computational **then**
6:    *R*_*sym*_←FetchRules(*P, G*_*KG*_)
7:    *V*_*calc*_, *T*_*trace*_←SymbolicEngine(*P, R*_*sym*_) ⊳ Deterministic Calculation
8:    *C*_*ctx*_←*T*_*trace*_
9:  **else**⊳ Definitional or Procedural
10:    *C*_*ctx*_←RetrieveContext(*P, G*_*KG*_) ⊳ Graph Retrieval
11:    *V*_*calc*_←Null
12:  **end if**
      **Phase 3: Multi-Modal Response Generation**
13:  *R*_*draft*_←LLM_Gen(*Q, V*_*calc*_, *C*_*ctx*_) ⊳ Generate Natural Language Explanation
14:  *R*_*final*_←ConsistencyCheck(*R*_*draft*_, *G*_*KG*_) ⊳ Verify against constraints
15:  **return** *R*_*final*_

### 3.1 Knowledge graph construction and refinement

The primary component of this methodology is a knowledge graph built from which taxes can be interpreted and computed. The Income Tax Act of 1961 is the legally recognized primary source on which this representation was created. In order to create this representation, the legal source was split into a hierarchy of sections, subsections, and clauses. The program was further extended to include pre-processing of the edited legal sections by removing any other formatting artifacts like headers, footers and page numbers, as well as normalizing any cross-references, e.g., “Section 10(10D)” has been converted to a concrete language model identifier. The normalization process was done in such a way that it created a unified structure of the Income Tax Act that is consistent with thousands of individual statutes within the Income Tax Act; this allowed the system to reason computationally about the law.

**Figure 3:**
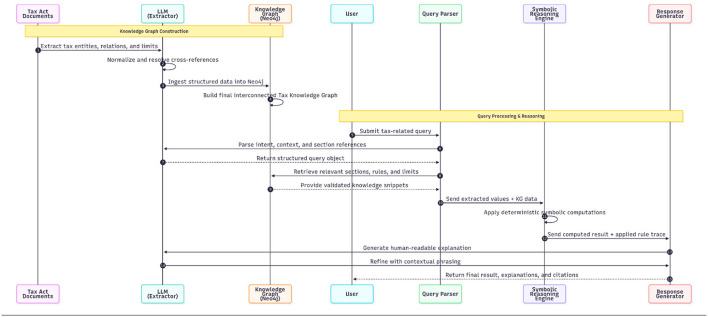
Sequence figure for data extraction.

The Normalized Legal Text represented a body of law that had been converted into an electronic standard. As shown in the sequence Figure 3 the extraction of structured legal knowledge from this content was accomplished using a large language model. This model extracted several entities from the normalized text, including taxpayers, deductions, exemptions and penalties, as well as the numerical limits and eligibility criteria for each. In addition, it created links between those entities and the underlying logic governing their application, excluding instances or creating dependencies. In essence, every entity was converted to base reasoning facts, for example, “Section 80C of the Income Tax Act provides a deduction for specified forms of investment to the actual deduction limit of 1.5 lakh rupees.” Each of those entities was represented in storage as “triples,” where each triple consisted of a subject-the entity, a predicate-the base fact and an object-the logical implication. The knowledge graph was created using these triples as the foundation.

To achieve semantic integrity and completeness, multiple refinement processes were conducted using cross-referencing extraction methods to correlate between portions of text that provided relevant context, the use of generated concept entities to support multi-hop reasoning across multiple domains which limited the number of nodes created so that the numeric extent could be treated as a first-order entity, and disambiguating entities to minimize the potential for entities to exhibit relatively high correlation in provision of similar or overlapping information. The numeric value assigned to basic facts extracted from the input text were converted to a standardized numeric representation, using the same reference as provided when describing probable associations between basic facts. Lastly, every fact that was extracted from the input text using the LLM was assigned a confidence score associated with the model's confidence level and the degree of consistency in fact extraction, to ensure conditional transparency and maintain adequate quality control.

**Figure 4:**
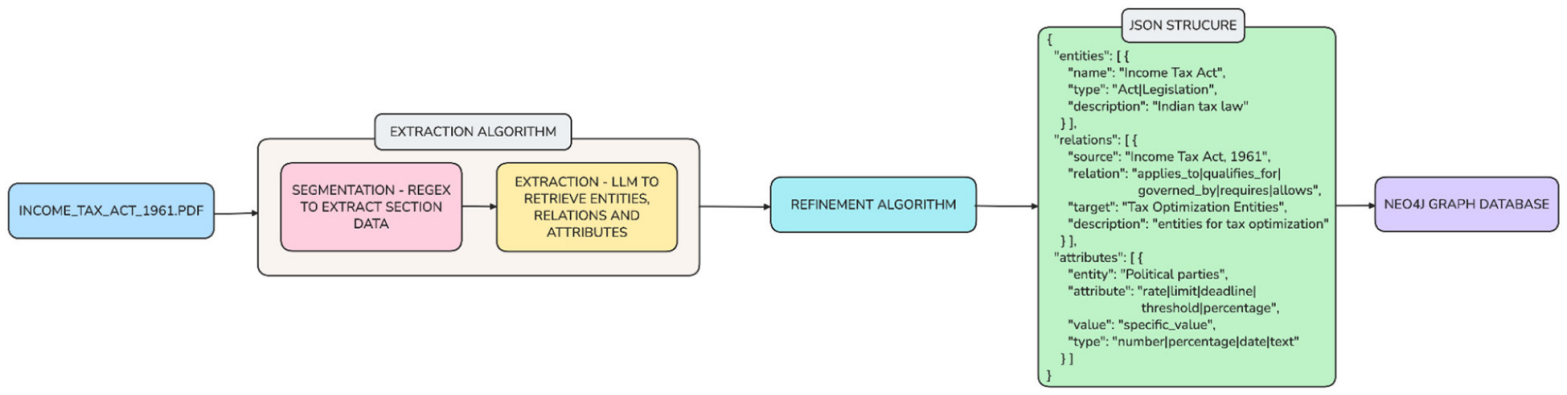
Knowledge graph construction architecture.

**Figure 5:**
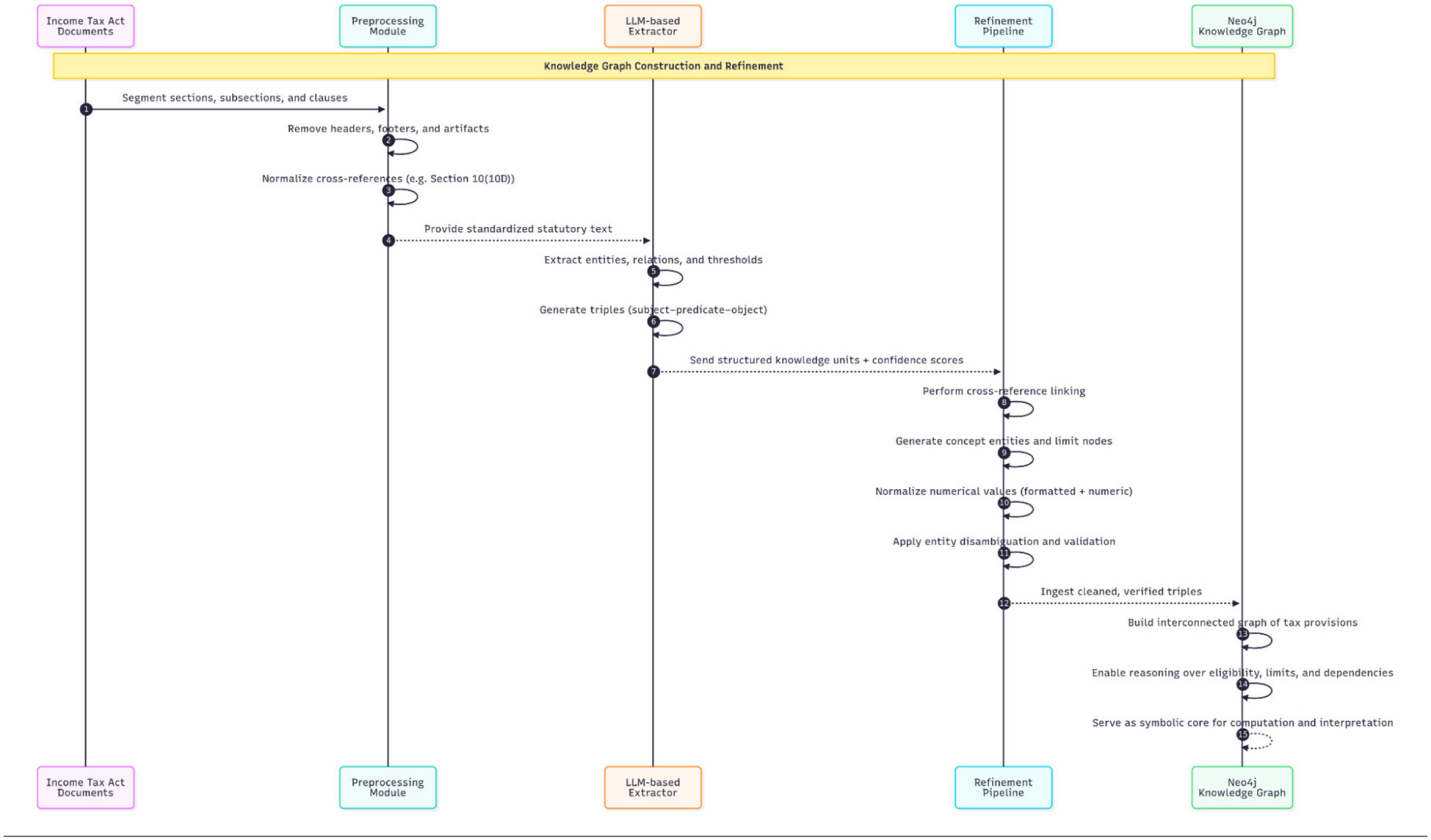
Sequence figure for graph construction.

That structured information, as shown in Figures 4, 5 was then used to create a Neo4j-based Knowledge Graph, which served as a comprehensive and interconnected map of tax provisions and relationships. The knowledge graph functions as the core of the reasoning engine, allowing the system to conduct sophisticated queries such as identifying whether a taxpayer is eligible for a particular deduction, confirming whether limits are applicable to that deduction, monitoring for cross-sectional dependency where tax provisions interact with each other, and assisting computation modules that directly link user-entered information to the associated legal provisions. By encoding the Income Tax Act in this interconnected manner, the system created a level of understanding and logical clarity that is unattainable with purely neural systems.

### 3.2 Hybrid query processing pipeline

**Figure 6:**
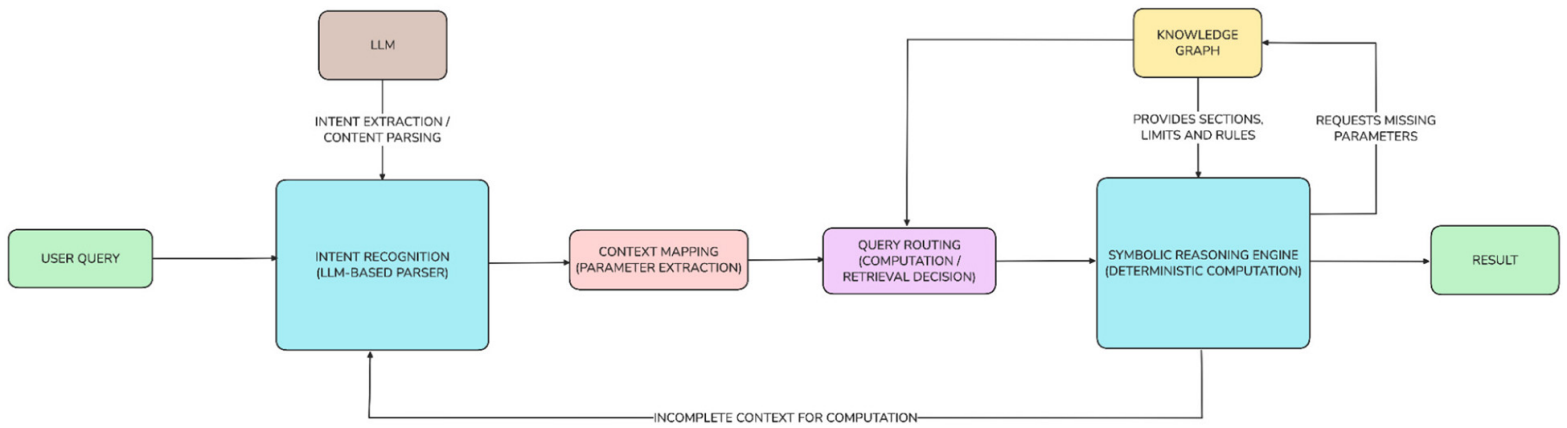
Query processing flow.

The query processing paradigm is the backbone of the solution, as the following components interact to produce accurate and interpretable tax computations: Natural Language Understanding, Structured Mapping, and Symbolic Reasoning. The architecture of the proposed solution is modular and includes four distinct sequential components: Intent Recognition, Context Mapping, Symbolic Reasoning, and Response Formatting. The functional separation of the components, as shown in Figure 6, allows each component to be optimized separately while maintaining the flow of relevant data from the user query through to the result generated by the system.

A user query may consist of an inquiry such as: “If my net profit is 12,00,000 rupees after disallowances, how much tax will I owe?” or “Am I eligible for a deduction in accordance with Section 80C?” The first step is for the system to determine intent through the use of a neural parser, which will assess whether the nature of the user inquiry is computational, eligibility, definitional, or procedural. The parser will also extract relevant context parameters including user type (individual, senior citizen, etc.), income type, age, financial year, and specifics of the user's investment(s). The result of this step will transform the user's original free form text into a structured representation that has been defined in a structured format like JSON, making it suitable for deterministic follow-on processing in the subsequent stages.

**Figure 7:**
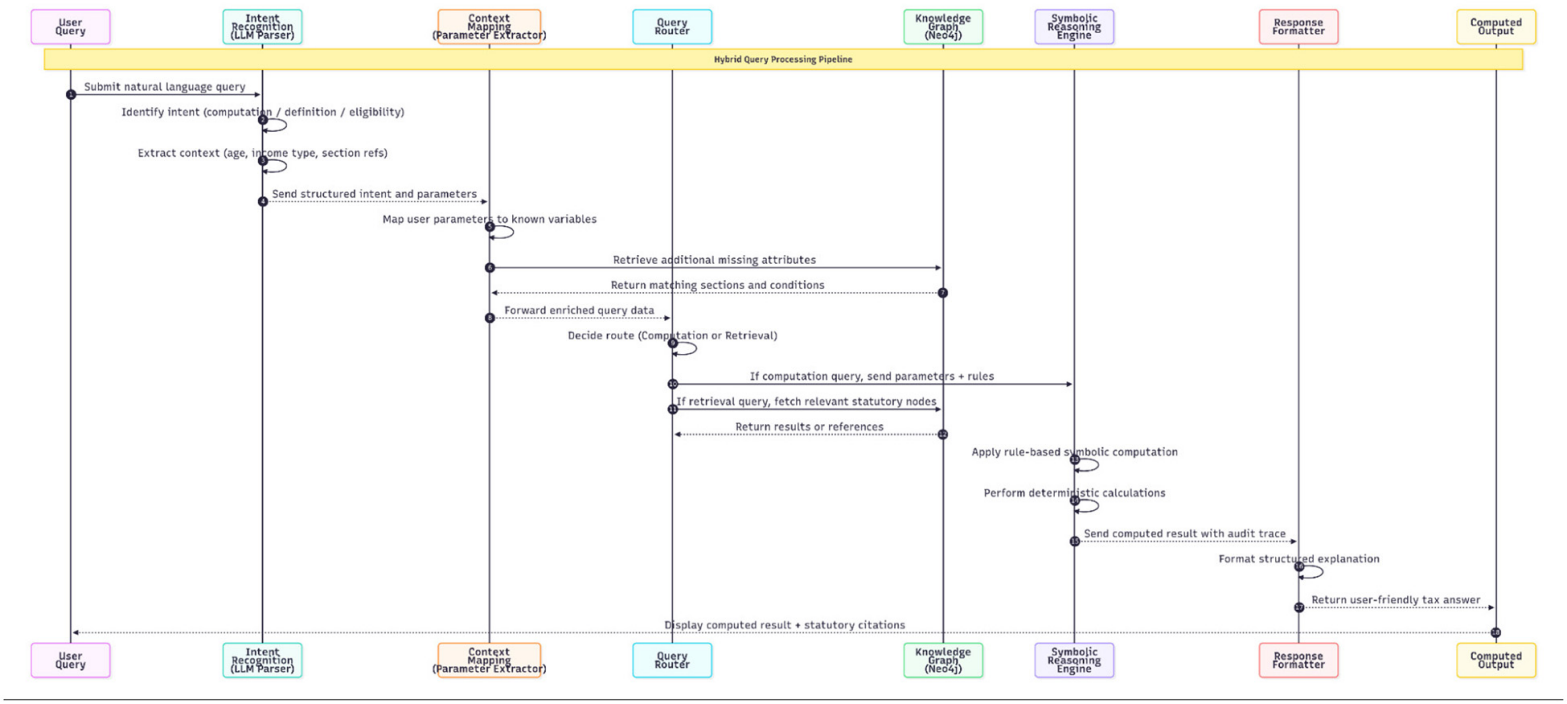
Sequence figure for query processing.

When a query's purpose and context are established, the software creates the appropriate logical pathway as shown in Figure 7. The queries that demand a computation only are directed to the symbolic reasoning engine, and the queries that require definitions or explanation will result in a retrieval of the relevant information from the knowledge graph. In the case that all of the necessary parameters were not supplied or clearly defined in the query, the system will make fallback retrievals from the knowledge graph as opposed to using incomplete computations as both of these tactics are critical to ensuring the accuracy and dependability of the system.

The symbolic reasoning engine is rule-based and strictly adheres to the Income Tax Act. The symbolic Reasoning Engine contains the logic for all the major income heads; i.e. Salary, House Property, Business and/or Profession, Capital Gains and Other Sources. Each rule has the tax law's logical definition for each income head, including the conditions, exemptions, and limits for each in the Act. For instance, to compute a business income all expense disallowances will be made and the full adjustment for depreciation will be made as provided in Section 32 of the Act. For Capital Gains all capital gains will be classified as either short-term or long-term with their applicable tax rates and exemptions for each classification. Similarly, Deductions Allowed in Chapter VI-A will be determined with checks for Mutual Exclusivity and Aggregation Limits.

Since there is no probabilistic reasoning in this global computational process, there are no hallucinations. All computations are made by and based on deterministic, rule-based logic with high precision numeric calculations. Each step can be seen through an audit trail. All inputs, all adjustments, all rules applied to inputs/adjustments, and final outputs to view transparently how each result was reached. Therefore, not only can the system calculate correctly, but also justify each answer under statutory authority.

### 3.3 Multi-modal response generation

The concluding phase of the methodology is the transmission of structured symbolic outputs into natural language for human interpretation while maintaining factual accuracy and computational transparency. The phase is accomplished by a multi-modal response generation layer that combines structured data formatting with neural, i.e., inference-based text generation. The purpose of this phase is to create a link between rigid computational processes and a form of communication that is as close to human readable as possible while allowing all forms of complex tax reasoning to remain credible and comprehensible to all users.

**Figure 8:**
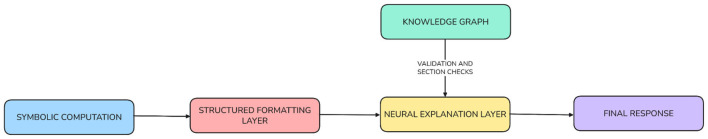
Response generation layer architecture.

The initial layer that generates response, as shown in Figure 8, focuses on generating the output in a defined format. The results generated by the symbolic computation which may include items such as computed taxable income, applied deduction amounts, applicable references and audit trails are generated into the accepted structure of results and also include supplementary metadata such as confidence scores, assumptions made, and warning messages, if relevant. Additionally, the structured results will allow for the integration of the output to tax filing systems, compliance dashboards or audit software; with all of these tools having the ability to programmatically verify each individual detail within the produced output.

**Figure 9:**
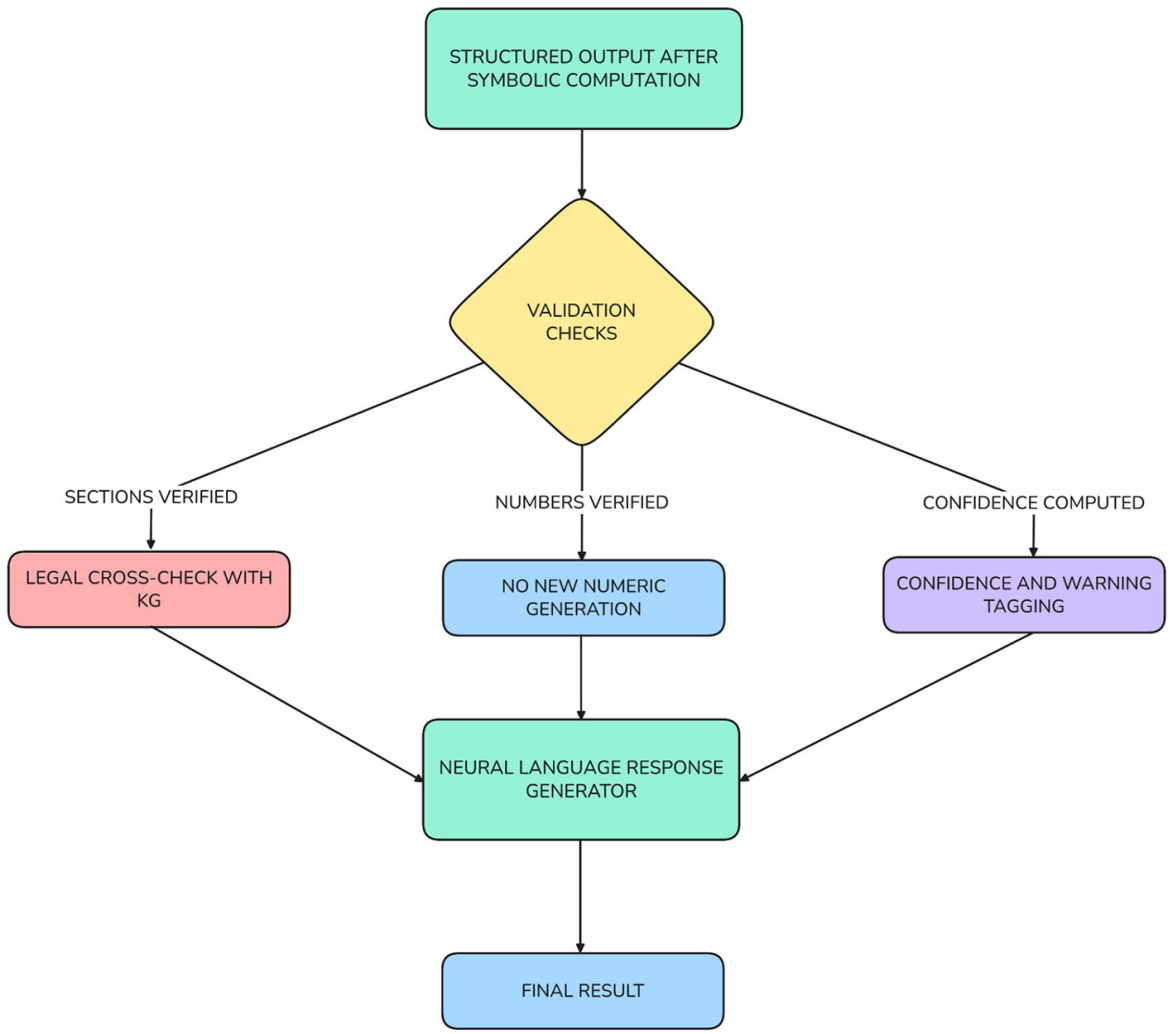
Response validation and hallucination control flow.

Layer two employs a neural-language explanation model that generates a natural language explanation for the output of the symbolic engine using recognized and approved output from the symbolic machine and knowledge graph. The explanation model translates the “tax result” to a natural-language summary for the user to understand the citations for the tax law that applies and the eligibility or ineligibility reasons with a semantic rationale for the entire process in “plain words.” Additionally, this creates multiple levels of controls to prevent confusion through hallucinations: the neural language model is not allowed to create or change numbers; the representations, as shown in Figure 9 are verified through the knowledge graph; and the explanations are only allowed to apply to the verified components of the system. The user is also provided with information on the con fidence levels for each of the responses.

The outcome of the two-layer combination results in a dual-layer system that produces accurate and understandable responses to the user, presented in a manner that is driven by user preferences. The explanations are easily identified back to their source, so that users whether professionals or non-professionals can be assured of the accuracy of the outcome as well as an understanding of how the outcome was determined. This dual-layer response process is a realization of the original vision of providing a combination of the logical accuracy of symbolic computing with the ease of use and improved communication provided by neural language models for a trustworthy human and tax compliance reasoning process.

## 4 Results and evaluation

### 4.1 Experimental setup

Local computer experimental testing was done using a desktop equipped with an NVIDIA GeForce RTX 3050 graphics card, Intel Core i5-12500 CPU, 16GB of RAM, and 1TB SSD. The NVIDIA GeForce RTX 3050 can provide a rapid method of inference for creating knowledge graph and performing any operations that require reliance on the GPU; the Intel Core i5-12500 provides support for the processing of data prior to use in the experimental data and for the operating system. The specifications of this computer provide sufficient computational resources for the production of valid and replicable experimental results, while delivering excellent operational efficiency.

Utilizing the uv package manager allowed for an orchestrated software environment capable of providing a high performance, reproducible virtual environment for all data processing activities. Legal PDF data was extracted using pdfplumber v0.11.4 and PyPDF2, maintaining the structural hierarchy present within the Income Tax Act. OpenAI's GPT-4o mini was used to build the Neuro-Symbolic integration for entity-relation extraction. Then, local semantic operations and deduplication were performed using the sentence-transformers library. The resulting knowledge graph was stored using Neo4j v5.0.0 in order to enable relational queries. Additionally, incremental JSON storage was employed to provide data continuity during the lengthy extraction processes. All experimental validations and unit tests were run against a pytest framework in order to validate the reliable handling of symbolic rules by the symbolic rule enforcement layer with respect to an accurate dataset of applicable tax regulations.

### 4.2 Evaluation metrics

An evaluation of the proposed neuro-symbolic tax optimisation engine used both quantitative performance metrics and qualitative reasoning evaluation criteria to assess the efficacy of the system within the domain of financial and legal reasoning tasks. In the quantitative analysis, four metrics, specifically accuracy, precision, recall and f1-score were used to compare performance on standardized classification/prediction tests (Equations 1–4). Accuracy represents the proportion of correct tax values computed by the system across the entire dataset used during evaluation, precision is the ratio of correctly predicted tax values to total tax values predicted by the system, recall is the ratio of correct tax values found by the system out of the total number of tax values expected, and f1-score represents the harmonic mean of both precision and recall, thus providing a balanced representation of overall performance under differing difficulties associated with tax computation query tasks.


$$\begin{array}{l} \text{Accuracy}=\frac{TP+TN}{TP+TN+FP+FN} \end{array}$$ (1)

$$\begin{array}{l} \text{Precision}=\frac{TP}{TP+FP} \end{array}$$ (2)

$$\begin{array}{l} \text{Recall}=\frac{TP}{TP+FN} \end{array}$$ (3)

$$\begin{array}{l} F1=\frac{2\times \text{Precision}\times \text{Recall}}{\text{Precision}+\text{Recall}} \end{array}$$ (4)

In this research project, a true positive (TP) is defined as a tax computation which produces a precise numerical result that is equal to and consistent with the statutory expected outcome for the given query. Furthermore,a correct answers metric has been compiled that specifies the total number of queries where the neuro symbolic tax optimization engine provided exact numerical equivalence of tax computed values to statutorily expected values out of 20 benchmark queries, this metric is particularly relevant in the context of tax computation as tax computations are deterministic legal tasks, a zero (0) tolerance for errors will be utilized in evaluating the system and therefore only exact numerical equivalence with the statutory outcome will be considered a true positive.

On their own, evaluating whether computer systems are accurately computing numerical value, i.e., numerical correctness does not constitute a complete evaluation when such systems are deployed in a legal-financial domain. Accordingly, additional qualitative elements were incorporated into the evaluation framework, as follows:

- **Legality of calculation pathways:** verification that each step of the computation aligns strictly with the computation provisions defined in the Indian Income Tax Act, 1961.
- **Semantic completeness:** determination of whether all relevant elements of a query are accounted for in the system's calculations, including implied statutory adjustments.
- **Reasoning consistency across multiple domains:** assessment of system performance across Business Income, Deductions, and Presumptive Taxation scenarios.
- **Explainability:** the system generates a natural language explanation of the reasoning process for the tax computation.
- **Traceability:** the system provides explicit references to the relevant sections of the Income Tax Act used in the computation pathway.

All of the qualitative attributes of the cases were assessed through the use of structured manual verification of the computation pathways to ensure that each step in the computation pathway could be traced back to a valid statutory provision and all of the elements of the query were adequately represented in the reasoning process. These evaluation metrics ensure that the framework captures not only numerical accuracy, but also legal completeness, reasoning robustness, and practical auditability of the systems under evaluation.

### 4.3 Experimental evaluation

The present segment involves an evaluation of the capabilities of the proposed Neuro-Symbolic Tax Optimisation Engine by contrasting it with two different baseline systems: a pure LLM only based system and a simple RAG based system. The intention of evaluating the performance of these two baseline systems relative to the performance of the Neuro-Symbolic Tax Optimisation Engine was to analyse not only the accuracy in terms of numerical similarity, but also examine the following attributes of financial reasoning and legal reasoning systems: Legality of the resulting solution as per the applicable Indian Income Tax Act,1961; Ability of the resulting solution to be accurate across varying scenarios of tax computation; Degree of Semantic Completeness of the resulting solution using linguistic analysis; Descriptive Abilities of the resulting solution.

To evaluate the system under realistic legal conditions, a benchmark of 20 expert validated, high-complexity tax scenarios were developed. These scenarios were purposefully developed to include edge cases where there are multiple—statutory dependencies, limits on deductions and cross-sectional interactions in the Income Tax Act which make them appropriate as “gold standard" reasoning benchmarks rather than large statistical data sets. The tax computation queries that were created reflect realistic tax-filing scenarios with varying levels of complexity including statutory caps, non-deductible expenses, recalculation of depreciation, presumptive taxation rules, etc. The curated benchmark dataset, as shown in Table 2 comprises multiple domains of taxation, i.e., Business Income Computation, Deductions under Section 80C and Section 80D, Presumptive Taxation under Section 44AD, etc. The inclusion of multiple domains requires use of both arithmetic-based methods and logic-based methods to arrive at a solution.

**Table 2:** Distribution of tax query difficulty across categories.

Category	Easy	Medium	Hard	Total	Description
Business income	1	4	3	8	Queries involving net profit adjustments, disallowed expenses, and depreciation calculations under various sections
Deductions (80C/80D)	7	3	0	10	Questions related to investment deductions, health insurance premiums, and statutory limits
Presumptive taxation	1	1	0	2	Scenarios involving Section 44AD calculations for small businesses
**Total**	**9**	**8**	**3**	**20**	Comprehensive coverage of Indian Income Tax Act provisions

The Table 3 provides a summary of various quantitative performance metrics for the three systems. The accuracy of each system is also represented in it using four standard measures: the overall system accuracy, i.e., accuracy, precision, recall, and F1-Score. From this perspective, it can be seen that the Neuro-Symbolic approach resulted in the highest overall accuracy of 80%, with the LLM-only being next highest at 75%, and the Simple RAG system at 60%. This represents an improvement over the baseline percentage for the LLM-only systems, especially given the limited number of evaluation records so that the effect of each inaccurate response is amplified in the overall percentage score across the three evaluations.

**Table 3:** Performance comparison of LLM-only, RAG, and neuro-symbolic tax reasoning systems.

Metric	LLM-Only	Simple RAG	Neuro-symbolic
Accuracy	75.0%	60.0%	**80.0%**
Precision	0.75	0.60	**0.80**
Recall	0.75	0.60	**0.80**
F1-score	0.75	0.60	**0.80**
Correct answers	15/20	12/20	**16/20**

A notable finding from the experiment was that the baseline RAG-model returned a lower, 60%, accuracy than the standalone LLM-based system, which returned 75% accuracy and a significant contributor to this result was the noise created in retrieval, itself, depending on how many “incorrect” or irrelevant documents were return in the retrieval. This could be due to the dense array of references and highly conditional statements found within legal statutes, such as the Income Tax Act, making it possible for retrieval algorithms to return provision that share some of the same wording. Also, when the retrieval output goes into the context of a prompt being sent to a model, it can make the model mislead or conflict with one or more pieces of the retrieved information which can negatively impacting a model's reasoning to reach an answer. In contrast, the standalone LLM-based system interprets tax scenarios based on the semantic representations generated/learned from within the pre-trained model but may incorrectly interpret tax scenarios and therefore arrive at an answer that do not match an actual scenario, thus giving rise to the “hallucinations” produced when generating the response. Because the proposed neuro-symbolic system combines the benefits of both of these systems through semantic extraction into the deterministic symbolic rule verification, we can achieve improved accuracies as well as improved consistency with legal statutes.

**Figure 10:**
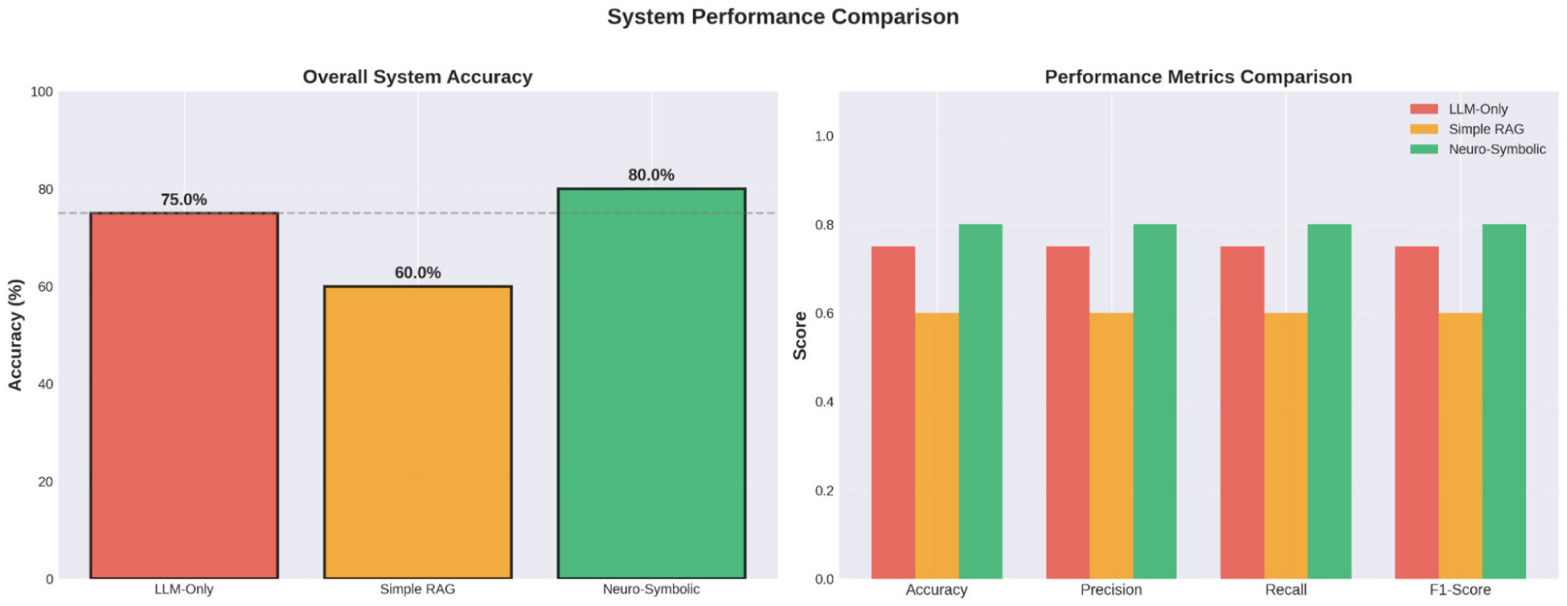
System performance comparison of the proposed model against benchmarks.

The analysis of numerical correctness at a more granular level, as shown in Figure 10 shows that the Neuro-Symbolic system consistently outperformed both baseline systems when faced with multi-step legal reasoning and arithmetic reconciliation. For example, business income computations requiring Permanent Add Backs (PAR) and Unallowed Expense Add Backs (AR) posed challenges to the LLM-only and Simple RAG baseline systems. Both baseline systems often failed to either properly AR unallowed expense amounts or misapplied the depreciation rules, i.e., either double counting or omitting adjustments. In contrast, the Neuro-Symbolic engine had a defined sequence of computations for how to compute taxable income, starting from net profit, applying statutory ARs and performing a gross-up calculation to determine allowable depreciation based on Written Down Value (WDV) and ultimately calculating taxable income. The results of the comparison of actual versus expected values for representative complex cases illustrated that the errors produced by baseline systems were often attributable to implicit assumptions and legal interpretation errors made by the systems, i.e., the systems did not have clear references to the law. The use of the deterministic symbolic layer within the proposed system alleviates this uncertainty so that each AR is legally sound and verifiable.

**Figure 11:**
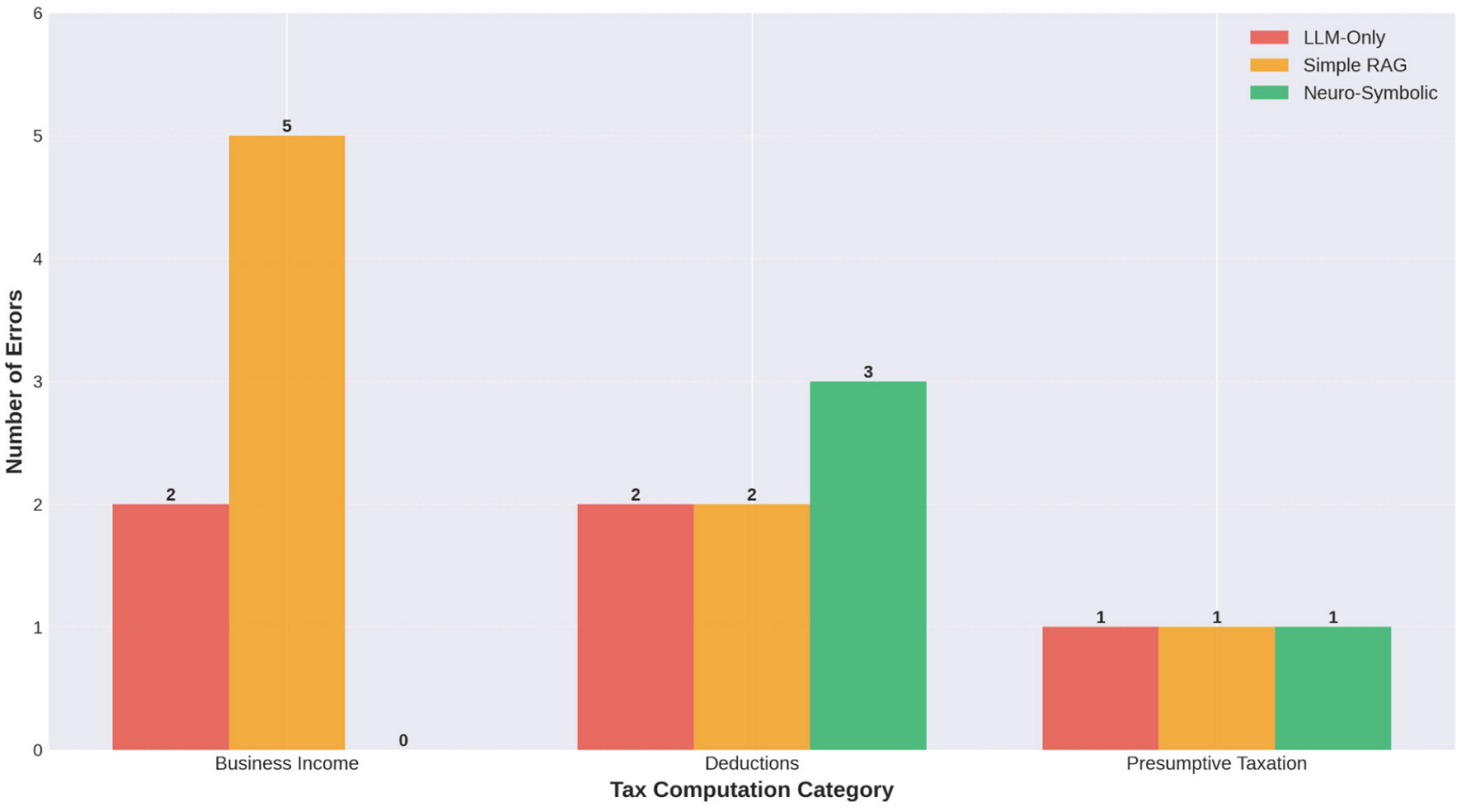
Category-wise error distribution.

The error analysis was conducted in three different tax domains, as shown in Figure 11, in order to develop a deeper understanding of the systems' behaviors. The analysis of errors, as shown in Table 4 indicated that there are clear differences in how each of the systems assigns errors to the three tax domains, based on the differences in the systems' computational, i.e., algorithmic and legal, i.e., statutory requirements. For example: the LLM-only system had a larger number of errors concentrated within the deduction-related questions related to statutory ceilings, e.g., Section 80D limit for senior citizens. In particular, the correct answer for these questions is a function of conditional thresholds rather than simply numerical arithmetic, resulting in a higher error rate for the LLM-only system. Conversely, the Simple RAG system exhibited a more flat and uniform distribution of errors across all three tax domains, indicating that errors associated with the Simple RAG system are likely due to the system's instability regarding retrieval relevance, i.e., inefficient inference matching and reasoning alignment, i.e., poor reasoned output alignment. In comparison, the neuro-symbolic system has a much lower overall error rate across all tax domains, with the remaining errors predominantly related to the incompleteness of the knowledge graph, rather than errors in the reasoning of the neuro-symbolic system. This finding indicates that the identified limitations of the neuro-symbolic system are predominantly structural, i.e., knowledge graph developing and can therefore be resolved through incremental expansion of the symbolic rules and graph entities. Through this analysis of the error distributions across the three tax domains, it becomes clear that the neuro-symbolic system is scalable and maintainable.

**Table 4:** Detailed comparison of system predictions across complex business income scenarios.

Query ID	Scenario description	Expected value (inr)	LLM-only predicted (inr)	Simple RAG predicted (inr)	Neuro-symbolic predicted (inr)	Correct systems
q1 business basic	Basic business with penalty and depreciation	12,20,000	11,40,000	12,20,000	12,20,000	RAG, Neuro-Symbolic
q2 business multiple disallowances	Multiple disallowances (IT, penalty, depreciation)	9,00,000	8,40,000	8,40,000	9,00,000	Neuro-Symbolic
q7 business complex	Multiple assets (machinery and building)	17,70,000	17,70,000	16,50,000	17,70,000	LLM-only, neuro-symbolic
q9 business vehicle	Delivery business with vehicles	14,70,000	15,30,000 *	15,70,000	15,30,000 *	LLM-only, neuro-symbolic
q13 business multiple assets	Large business with penalty and multiple assets	24,60,000	23,60,000 *	21,10,000	23,60,000 *	LLM-only, neuro-symbolic
q17 business it paid	IT paid and depreciation adjustments	16,80,000	16,80,000	17,70,000	16,80,000	LLM-only, neuro-symbolic
q19 business mixed	Mixed disallowances (fine, IT, depreciation)	22,85,000	21,75,000 *	21,75,000 *	22,85,000	All (neuro-symbolic exact)

**Figure 12:**
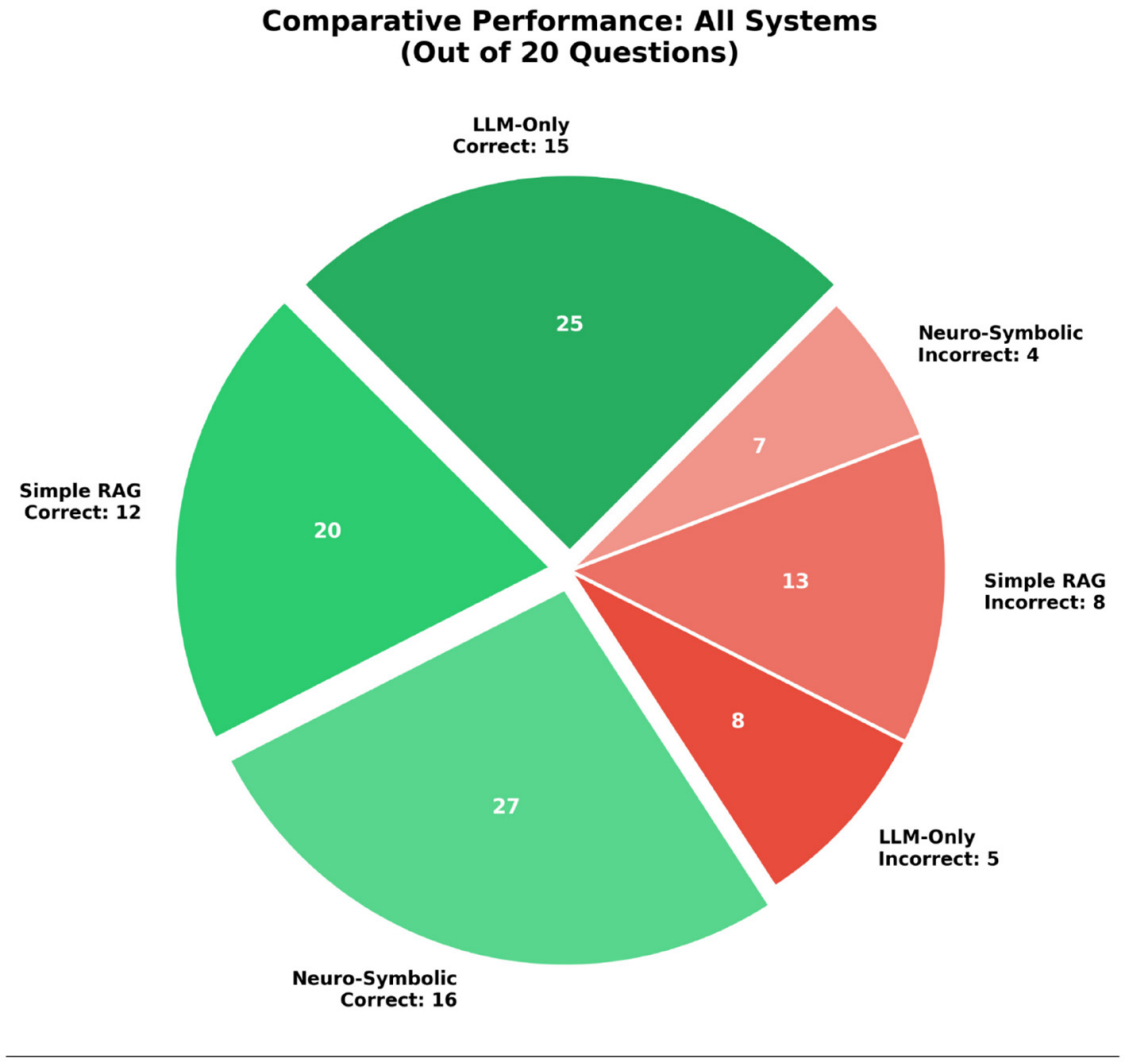
Comparative performance.

The determination of the qualities of “explainability” provides insight into further advantages presented by the Neuro-Symbolic architecture used. The Neuro-Symbolic architecture generates “structured computation traces” which outline incremental components used to arrive at each answer based on evidence from the query. An example of this would be “disallowed expense AR calculations” that are clearly presented on a page as part of the structured computation trace's presentation as per the performance. It should be noted that each part of the structured computation trace can be directly associated with a specific section of the Income Tax Act, thus allowing them to be easily verifiable by an auditor or tax professional. In contrast, the narratives generated from the LLM-only and Simple RAG systems which were primarily composed of prose were generally missing a clear presentation of the intermediate values or clear connections to legal provisions as shown in Figure 12. Because of this, it is difficult to ensure the verifiability of these systems in practice, since they do not provide the necessary traceability for each output value/output source, and as such, cannot withstand the level of scrutiny currently required for any tax values in the industry. The Neuro-Symbolic architecture's capacity to generate fully auditable, detailed step-by-step reasoning represents a momentous step toward achieving confidence in the deployment of AI systems to meet legislative requirements.

### 4.4 Evaluation dataset

The dataset used for evaluation included 20 complex tax scenarios that matched the requirements needed to assess an individual's ability to perform high-complexity advisory queries based on the Indian Income Tax Act, 1961. Each of these tax scenarios required reasoning over multiple statutory provisions, deduction rules, and eligibility criteria as opposed to simple single-rule queries. The evaluation dataset was intentionally designed to serve as a high-complexity benchmark to measure reasoning accuracy and statutory compliance, rather than as a statistically significant dataset with large sample sizes. Each of the queries in the evaluation accessed different parts of the knowledge graph and the symbolic rule engine and included multi-step reasoning in order to simultaneously measure the semantic interpretation, deterministic computation, and legal validity of the entire evaluation data set. Thus, the evaluation dataset is reflective of the real world tax advisory environment, in which a much smaller number of complex cases is frequently a good indicator of system performance compared to a much larger number of simple queries.

### 4.5 Validation procedure

Authors conducted a manual verification process to verify qualitative evaluation metrics i.e., semantic completeness and reasoning consistency of a generated tax computation against relevant provisions of the Income Tax Act. Every system output was cross-referenced to ensure the deductions, exemptions and final calculations adhered to the applicable rules represented in both the Knowledge Graph and symbolic reasoning layer to maintain compliance with the legal framework encoded within the system.

### 4.6 Failure mode analysis

Despite the Neuro-Symbolic Tax Optimisation Engine having an average predictive accuracy of 80%, an error analysis was completed to investigate why certain predictions were wrong. Errors mainly came from three categories: (1) missing rules in the knowledge graph; (2) ambiguity in the interpretation of legislation; and (3) complex multiple clause deductions where rules interact. The most common reason for getting predictions wrong was that the rules on taxation were not completely covered in the knowledge graph. Certain parts of the Income Tax Act (ITA) contain nested clauses and reference each other, resulting in not having a complete representation of the rules in the graph. Thus the symbolic reasoning engine could not resolve the constraints it found regarding the application of the rules.

Another reason for incorrect predictions was that some sections of tax legislation were ambiguous in terms of their meaning. There were instances where there were overlapping clauses of taxation where there could be several deductions and/or exemptions that could be claimed at the same time. The symbolic reasoning engine will always apply the same calculations due to the deterministic nature of the symbolic reasoning, but some of the entities extracted from the data could be ambiguous, which resulted in incorrectly selecting tax rules. There was a small number of incorrect predictions made due to the complexity of the scenarios involving multiple types of deduction and exemption, which all have interactions with each other. The complexity of these tax scenarios created multi-hop reasoning through many different knowledge graph nodes, which increased the chance of missing intermediate dependencies. Observational analysis demonstrates that the primary issue observed was limited knowledge graph coverage instead of a problem with the symbolic reasons engine itself. Expanding the statutory knowledge graph, along with refining the entity extraction pipelines should allow for continued improvement of system performance as future iterations are developed.

## 5 Conclusion

The initial development effort needed to create a symbolic knowledge base is one challenge of a neuro-symbolic system. Creating an accurate knowledge graph of the Income Tax Act will require careful extraction of entities, relationships, and statutory rules and may require collaboration between technical developers and experts in the field. Once the knowledge graph and symbolic rule set have been established, however, the architecture will allow for structured updates and better long-term maintenance. The semi-automated process of extracting entities using large language model techniques has reduced the amount of manual effort required during the construction of the knowledge graph in this study.

In addition, continuing maintenance will need to be performed to incorporate any changes or updates to applicable federal tax law, which typically occur through annual budgets as well as by way of taxed laws that are revised at some point during the fiscal year. This requires automated systems to be flexible enough to accommodate these continual changes. To mitigate this problem, the proposed architectural structure separates statutory components from the neural components. By doing so, changes to the symbolic rules can be made in the symbolic rule layer and nodes can be updated in the knowledge graph without having to retrain any of the machine learning models used to create those nodes and rules, thereby avoiding any expensive computations.

In contrast to single, independent large language models, a hybrid architecture requires several additional processing stages before generating a response (i.e., knowledge base retrieval and formal rules verification). As a result, the response from a hybrid architecture may take longer than an individual LLM generating a response directly. However, the hybrid architecture design emphasizes the importance of producing correct and traceable responses as well as conforming to applicable governing laws and regulations versus generating a response as quickly as possible. In the areas of taxation and legal advice, for example, an incorrect response can result in a large financial or regulatory penalty. Therefore, the improved reliability, robustness and improved explainability of a neuro-symbolic approach outweigh the minimal increment in computational resources required by the hybrid architecture.

A major advantage of an automated tax reasoning system is the ability to support non-static statutory changes, including those resulting from new tax legislation each year. The proposed neuro-symbolic architecture provides a solution to this problem by being modular in structure. The tax code's statutory knowledge is maintained as an external structure in a structured tax knowledge graph rather than being built into weight matrices within the neural network. Therefore, modifications and additions to statutory tax provisions can be accomplished simply by modifying or adding nodes, relationships and rule definitions within the tax knowledge graph. Consequently, this design enables the ability to support incrementally updating the statutory portions of the architecture without having to re-train the neural parts of the architecture. This modularity and use of structured graphical representation allows for tax reasoning systems to be highly adaptable, logically consistent and auditable as tax laws continue to change over time.

The data generated from this experiment conclusively indicate that the Neuro-Symbolic Tax Optimisation Engine performed at an 80% rate, higher than either the LLM-only baseline (75%) or the simple RAG system (60%) across a variety of benchmark tax computation scenarios. We show that combining immutable rules in the form of symbols as ground truth with LLMs or other forms of semantic interpretation greatly reduces the number of common failure modes found in resources such as hallucinations, arithmetic inconsistencies, and the application of inappropriate statutory provisions. The architecture presented in this work has been implemented in a manner where the symbolic reasoning component functions as a regulatory guardrail to ensure that the probabilistic outputs of the language model remain within the limitations of the deterministic statutory rules. Although the models based on LLMs and Simple RAG systems are able to produce fluent output in terms of human language, neither is able to provide the determinism and traceability needed to support the computation of tax liability. The results of this research provide empirical evidence for the core thesis of this work: that a hybrid model of neuro-symbolic reasoning is a more appropriate and trustworthy framework from which to build tax automation solutions within legally constrained financial environments.

While the outcomes of this study are promising, there are limitations to the work presented in this study. One of the limitations is maintaining the accuracy of the representation of the knowledge graph, which will require continuous updates as legislation changes through amendments and policies. More research in the future will be necessary to explore the establishment of automated pipelines to maintain symbolic knowledge graphs directly from legislative documents and government gazettes to ensure long-term scalability and regulatory compliance.

While the focus of this research is on tax calculations as proscribed in the Indian Income Tax Act, the neuro-symbolic architecture proposed in this research also has applications in other high-stakes decisions where regulatory compliance and explainability are a requirement. Examples of these kinds of high-stakes decision environments include; (i) medical compliance systems, (ii) insurance underwriting, (iii) financial audit, and (iv) environmental regulatory analysis. The proposed neuro-symbolic architecture can potentially provide a framework for integrating probabilistic language capabilities with deterministic regulatory enforcement.

Therefore, this research provides a foundational blueprint for creating legally-aware autonomous systems that can function reliably and effectively in complex regulatory environments by bridging the gap between linguistic flexibility and statutory rigidity.

## Data Availability

The raw data supporting the conclusions of this article will be made available by the authors, without undue reservation.

## References

[B1] Adhikary S. Roy D. Ghosh K. Sen P. (2026). A case study for automated attribute extraction from legal documents using large language models. Artif. Intell. Law 34, 245–266. doi: 10.1007/s10506-024-09425-7

[B2] Al Zubaer A. Granitzer M. Mitrović J. (2023). Performance analysis of large language models in the domain of legal argument mining. Front. Artif. Intell. 6:1278796. doi: 10.3389/frai.2023.1278796 38045763 PMC10691378

[B3] Bhuyan B. P. Ramdane-Cherif A. Tomar R. Singh T. P. (2024). Neuro-symbolic artificial intelligence: a survey. Neural Comput. Appl. 36, 12809–12844. doi: 10.1007/s00521-024-09960-z

[B4] Cunnington D. Law M. Russo A. Lobo J. (2023). FFNSL: feed-forward neural-symbolic learner. Mach. Learn. 112, 515–569. doi: 10.1007/s10994-022-06278-6

[B5] García-Barragán Á. Sakor A. Vidal M. E. Menasalvas E. Gonzalez J. C. S. Provencio M. . (2025). NSSC: a neuro-symbolic AI system for enhancing accuracy of named entity recognition and linking from oncologic clinical notes. Med. Biol. Eng. Comput. 63, 749–772. doi: 10.1007/s11517-024-03227-4 39485651 PMC11891111

[B6] Gogani-Khiabani S. Tizpaz-Niari S. Trivedi A. Chyi S. P. (2025). Performance of LLMs on VITA test: potential for AI-assisted tax returns for low income taxpayers. Artif. Intell. Law 1–22. doi: 10.1007/s10506-025-09465-7. [Epub ahead of print].

[B7] Górski Ł. Kuźniacki B. Almada M. Tyliński K. Calvo M. Asnaghi P. M. . (2025). Exploring explainable AI in the tax domain. Artif. Intell. Law 33, 551–579. doi: 10.1007/s10506-024-09395-w

[B8] Lorello L. S. Lippi M. Melacci S. (2025). The KANDY benchmark: incremental neuro-symbolic learning and reasoning with Kandinsky patterns. Mach. Learn. 114. doi: 10.1007/s10994-025-06798-x. [Epub ahead of print].

[B9] Pahsa A. (2024) Financial technology decision support systems. J. Electr. Syst. Inf. Technol. 11:5. doi: 10.1186/s43067-023-00130-0.

[B10] Saba W. S. (2023) “Stochastic LLMs do not understand language: towards symbolic, explainable and ontologically based LLMs,” in Lecture notes in computer science. 14320 LNCS, eds. Almeida J. P. A. Borbinha J. Guizzardi G. Link S. Zdravkovic J. (Cham: Speinger), 3–19. doi: 10.1007/978-3-031-47262-6_1.

[B11] Saragih A. H. Reyhani Q. Setyowati M. S. Hendrawan A. (2023). The potential of an artificial intelligence (AI) application for the tax administration system's modernization: the case of Indonesia. Artif. Intell. Law 31, 491–514. doi: 10.1007/s10506-022-09321-y

[B12] Spillo G. Musto C. Gemmis M. d. Lops P. Semeraro G. (2024). Recommender systems based on neuro-symbolic knowledge graph embeddings encoding first-order logic rules. User Model. User-Adapt. Interact. 34, 2039–2083. doi: 10.1007/s11257-024-09417-x

[B13] Tong R. J. Hu X. (2024) Future of education with neuro-symbolic AI agents in self-improving adaptive instructional systems. Front. Digit. Educ. 1, 198–212. doi: 10.1007/s44366-024-0008-9.

[B14] Waiker V. Gohokar H. Alazzam M. B. Sulaiman S. F. Gupta D. Bala B. K. (2025). “Predicting tax defaults through feature transformation and XGBoost optimization,” in Proceedings of 2025 3rd international conference on intelligent systems, advanced computing, and communication, ISACC 2025 (Silchar), 374–379. doi: 10.1109/ISACC65211.2025.10969365

[B15] Wei B. Yu Y. Gan L. Wu F. (2025). An LLMs-based neuro-symbolic legal judgment prediction framework for civil cases. Artif. Intell. Law. doi: 10.1007/s10506-025-09433-1. [Epub ahead of print].

[B16] Yang Y. Chen J. Xiang Y. (2024). A review on the reliability of knowledge graph: from a knowledge representation learning perspective. World Wide Web. 28. doi: 10.1007/s11280-024-01316-w

[B17] Yang Z. Yuan S. Li W. Shao Z. Liu R. (2025). A review on synergizing knowledge graphs and large language models. Computing 107, 1–25. doi: 10.1007/s00607-025-01499-8

[B18] Yu J. McCluskey K. Mukherjee S. (2020). “Tax knowledge graph for a smarter and more personalized TurboTax,” in International workshop on knowledge graph.

[B19] Zhong Y. Wong D. Lan K. (2024). Tax intelligent decision-making language model. IEEE Access. 12, 146202–146212. doi: 10.1109/ACCESS.2024.3419079

[B20] Zhu X. Liu B. Yao L. Ding Z. Zhu C. (2023). TGR: neural-symbolic ontological reasoner for domain-specific knowledge graphs. Appl. Intell. 53, 23946–23965. doi: 10.1007/s10489-023-04834-8

